# Value of the arterial stiffness index and ankle brachial index in subclinical atherosclerosis screening in healthy community-dwelling individuals

**DOI:** 10.1186/s12889-019-6398-9

**Published:** 2019-01-15

**Authors:** Javad Alizargar, Chyi-Huey Bai

**Affiliations:** 1 School of Public Health, College of Public Health, Taipei Medical University, 250 Wu-Hsing Street, Taipei City, 11031 Taiwan; 2 Department of Public Health, College of Medicine, Taipei Medical University, 250 Wu-Hsing Street, Taipei City, 11031 Taiwan

**Keywords:** Carotid intima media thickness, Atherosclerosis, Arterial stiffness, Ankle brachial index, Carotid artery plaque

## Abstract

**Background:**

Carotid intima media thickness (cIMT) and the carotid plaque score (cPS) are valid markers for detecting subclinical atherosclerosis. Evaluation of ASI and ABI for detection of atherosclerosis is assessed in this study. Finding a model to see which individual has a risk of having atherosclerosis, so those people can be further assessed by invasive but more accurate atherosclerosis detection methods like angiography is another objective of this study.

**Methods:**

Data of 212 healthy community-dwelling subjects, consisting of carotid duplex records, ASI and ABI measurements, certain laboratory tests, and related cardiovascular disease (CVD) risks were analyzed for correlations.

**Results:**

The ABI was independently associated with high cPS. Age, hypertension and Waist circumference are determinants of subclinical atherosclerosis as in high cIMT and high cPS.

**Conclusions:**

The use of the ASI cannot replace carotid ultrasound in detecting subclinical atherosclerosis because it is not independently associated with high cIMT and cPS while ABI can be used in detection of high cPS in healthy community-dwelling individuals. Public health policies to encourage weight reduction and treating hypertension can help prevention of subclinical atherosclerosis in healthy community-dwelling individuals. Models consist of age, body compositions like waist circumference and hypertension history can be used in further assessment of atherosclerosis.

## Background

Atherosclerosis is a chronic process that involves arterial walls and remains silent until some critical event like stenosis occurs; thus, it is the underlying process of cardiovascular (CV) disease (CVD) and cerebrovascular disease and death [1]. Atherosclerosis is a long process before the plaques that have formed cause severe stenosis or rupture [2], so early detection and screening for subclinical atherosclerosis is important in preventing CVD.

The carotid intima media thickness (cIMT) and measuring the carotid plaque score (cPS) in the carotid artery were identified as valid screening methods for CVD. Criteria of > 75 percentiles of cIMT and cPS values adjusted for age and sex were identified as risk factor adjuncts to traditional risk factors for CVD [3]. An increased cIMT and the presence of carotid plaques are correlated with CVD in older and even some younger patients [4, 5]. The cIMT and cPS are valid markers of subclinical atherosclerosis as respectively presented in early- and late-stage subclinical atherosclerosis [6], but more invasive methods like angiography remains the gold standard for the diagnosis of arterial disease.

The ankle-brachial index (ABI) is an effective, simple, and noninvasive method to screen and diagnose peripheral artery disease (PAD) and has a high sensitivity and specificity and small variability [7]. American Heart Association guidelines suggest the ABI as a screening tool for people with PAD in subjects older than 65 years, or with a risk factor (hypertension, smoking, diabetes mellitus (DM), or dyslipidemia) and a family history of PAD and people with any known form of atherosclerosis, and also suggest the ABI as a good tool for CVD risk assessments [8]. The ABI is correlated with atherosclerosis and can predict CV death and all-cause mortality [7, 9, 10].

Clinically, the ASI is the most commonly used concept to describe large arteries properties and can provide a quantitative measure of atherosclerosis [2]. The ASI was suggested to be used as a screening test for CVD and atherosclerosis risk in asymptomatic subjects [11]. Calculation of the ASI with Cardiovision MS-2000® was proven to be correlated with other methods of arterial stiffness like the pulse wave velocity (PWV) and also with coronary artery disease (CAD) [12].

The ABI, ASI, and cIMT are screening tests for atherosclerosis [11]. But relationships between these tests and factors associated with subclinical atherosclerosis in healthy community-dwelling individuals have not been studied in detail in the literature so far. The study objectives were to determine the value of the ASI and ABI in subclinical atherosclerosis. We also tried to find a model which uses the clinical and laboratory information and atherosclerosis risk factors to see which patient has a risk of having atherosclerosis, so those people can be further assessed by invasive but more accurate atherosclerosis detection methods like angiography. Different independent determinants of subclinical atherosclerosis were also of our interest.

## Methods

**Fig. 1:**
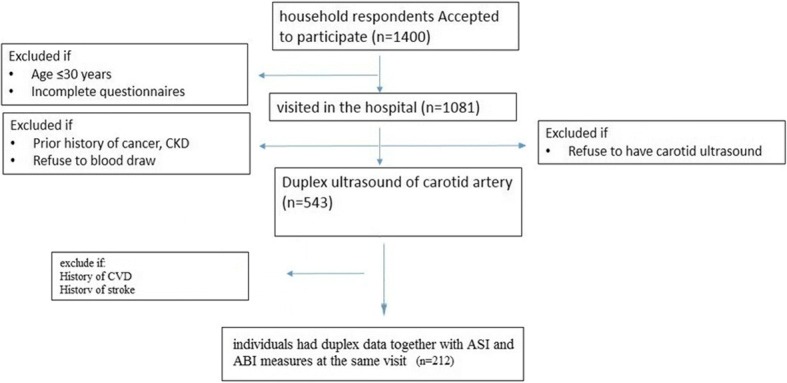
Flowchart for selection of the study participants. CKD: Chronic Kidney Disease; CVD: Cardiovascular Disease

We used data from a community-based prospective cohort study that was designed to evaluate the CV and cerebrovascular risk factors in individuals. The detailed method of participant selection been explained elsewhere [13, 14]. In summary, from 543 individuals which had the duplex ultrasound, people without prior history of CVD and stroke were invited for ASI and ABI measurement. Data of Individuals who had complete ASI, ABI, duplex ultrasound measurements and laboratory results were analyzed for this study. Flowchart of this study can be found in Fig. 1. All study subjects signed a consent form in order to enter the original study, and no names were revealed in the results. The study received full ethical approval from Taipei Medical University with Institute Review Board (IRB) reference numbers 94E-183, 94E-198 and 96E-004.

Based on Receiver operating curve (AUC) of 0.644 for prediction of plaque formation with ASI, reported by Kao et al. [2], with sample size of more than 141 we can have statistical power more than 80 (alpha error = 0.05, null hypothesis value = of 0.5, ratio of sample sizes in negative / positive groups = 2) [15]. Further power calculation will be done on the Pearson’s correlations between cIMT, cPS and ASI and ABI in the case of significance correlation using G Power 3.1.9.2 software. Simple and descriptive statistics were used as numbers and percentages for categorical variables and the mean and standard deviation (SD) for continuous variables. Fisher’s exact test was used for categorical variables based on age group (< 45, 45~60, and > 60 years) and sex. Analysis of variance (ANOVA) test was used for continuous variables. Data including demographic characteristics, such as sex, age (years), height (cm), weight (kg), waist circumference (WC; cm), bottom circumference (BC; cm), body-mass index (BMI; kg/m^2^), basal metabolic rate (BMR, calories) body fat (BF, %), a history of type 2 (T2) DM, a history of hypertension (HTN), cigarette smoking (Smk), perceived stress level (Stressed), alcohol consumption, systolic (SBP) and diastolic blood pressure (DBP) (mmHg), and pulse (beats/min). The questionnaire had six perceived stress levels: very high, high, moderate, low, very low, and none. An individual was considered stressed if he/she answered high or very high. An individual was considered to consume alcohol if he/she drank more than three times a week, and more than 2 cups each time. Those who had ever smoked in their life were considered a smoker.

Laboratory data, including uric acid level (URCA, mg/dl), fasting blood sugar (FBS, mg/dl), creatinine (Cr, mg/dl), blood urea nitrogen (BUN, mg/dl), aspartate aminotransferase (AST, units/L), alanine transaminase (ALT, units/L), cholesterol (Chol, mg/dl), triglycerides (TGL, mg/dl), low-density lipoprotein (LDL, mg/dl), high-density lipoprotein (HDL, mg/dl), glycated hemoglobin (HBA1c; %) and high-sensitivity C-reactive protein (CRP, mg/L) were gathered using standard methods (using an X-1500-Sysmex, Germany, Beckman AU5800, USA, and Tosoh HLC-723G8 automated glycol-hemoglobin analyzer, Japan).

The ASI and ABI was assessed using an MS-2000 model (International Medical Devices Partners, Las Vegas, NV, USA). An oscillometric method was used by the device, and its cuffs have sensors that transmit data to the device. After 20 min of rest, a subject was placed in a supine position, and the cuffs were wrapped around the extremities. The device provides data on the AS, ABI, SBP, and DBP, according to the arterial volume pattern changes caused by the steady decrease in cuff pressure after 9 min. The ABI was automatically calculated by the device as the ankle SBP/brachial SBP ratio. The device used the higher value of the brachial SBP between the right and left arms. The left and right ABIs were calculated, and a mean ABI value was used as a parameter in our study. An ABI of < 0.95 was considered a low ABI (LABI). The BF and BMR were calculated by a bioelectrical impedance method using an Omron Body Fat Analyzer HBF-306 device, Japan. The BMI was calculated by dividing the weight in kg by the height in meters squared.

Carotid and vertebral arteries ultrasound was conducted using a B-mode Duplex ultrasound (SONOS 5500, HP, USA). Peak systolic velocity (PSV; cm/s), flow (ml) and end diastolic velocity (EDV; cm/s) measured in one cardiac cycle for every individual. The resistance index (RI) and mean velocity (MV) were calculated using these respective formulas: RI = (PSV-MV)/PSV and MV = (PSV + 2*EDV)/3. Diameters of the internal carotid artery (ICA) beyond the carotid bulb, the cervical portion of the common carotid artery (CCA), the external carotid artery (ECA), vertebral artery (VA) sequentially on both sides were measured. The number of plaques in the carotid arteries were measured on each side by a cardiologist. The cPS in each person was the sum of the numbers of plaques on the right and left sides.

The cIMT assessment was done at 1 cm distance from the carotid bulb on the left and right sides. The mean cIMT and diameter of each artery were calculated by taking the average of the left and right sides in each individual. The averages of all flows and RIs of the left and right carotid and vertebral arteries was calculated for obtaining the mean flow and RI for every subject.

The levels of 75% quartile of all subjects’ cIMT and cPS data were used as the cutoff points for the thick cIMT and high cPS. A simple Pearson’s correlation was calculated for the cIMT, cPS, ASI and ABI with other variables in our study. Variables with significant correlations with the cIMT and cPS, along with the demographic variables, were included in a multiple linear regression to detect high cIMT and cPS. Variables that calculated the same concept or had a high collinearity were not included in the model at the same time, and the one with the highest correlation was chosen for inclusion in the model. The ASI and ABI was not put in the models, as we used them for screening properties for subclinical atherosclerosis. Multiple linear models were run for detecting the associations of the ASI and ABI with the cIMT and cPS.

Two logistic regressions were conducted using variables with a significant correlation with the cIMT and cPS in the previous models to calculate the odds ratio (OR) and 95% confidence interval (CI) for observing the association. Observations were deleted due to missing values for the response or explanatory variables in all models. The alpha error was set to 0.05, and we used SAS vers. 9.4 (SAS, Cary, NC, USA) for all data analyses.

## Results

**Fig. 2:**
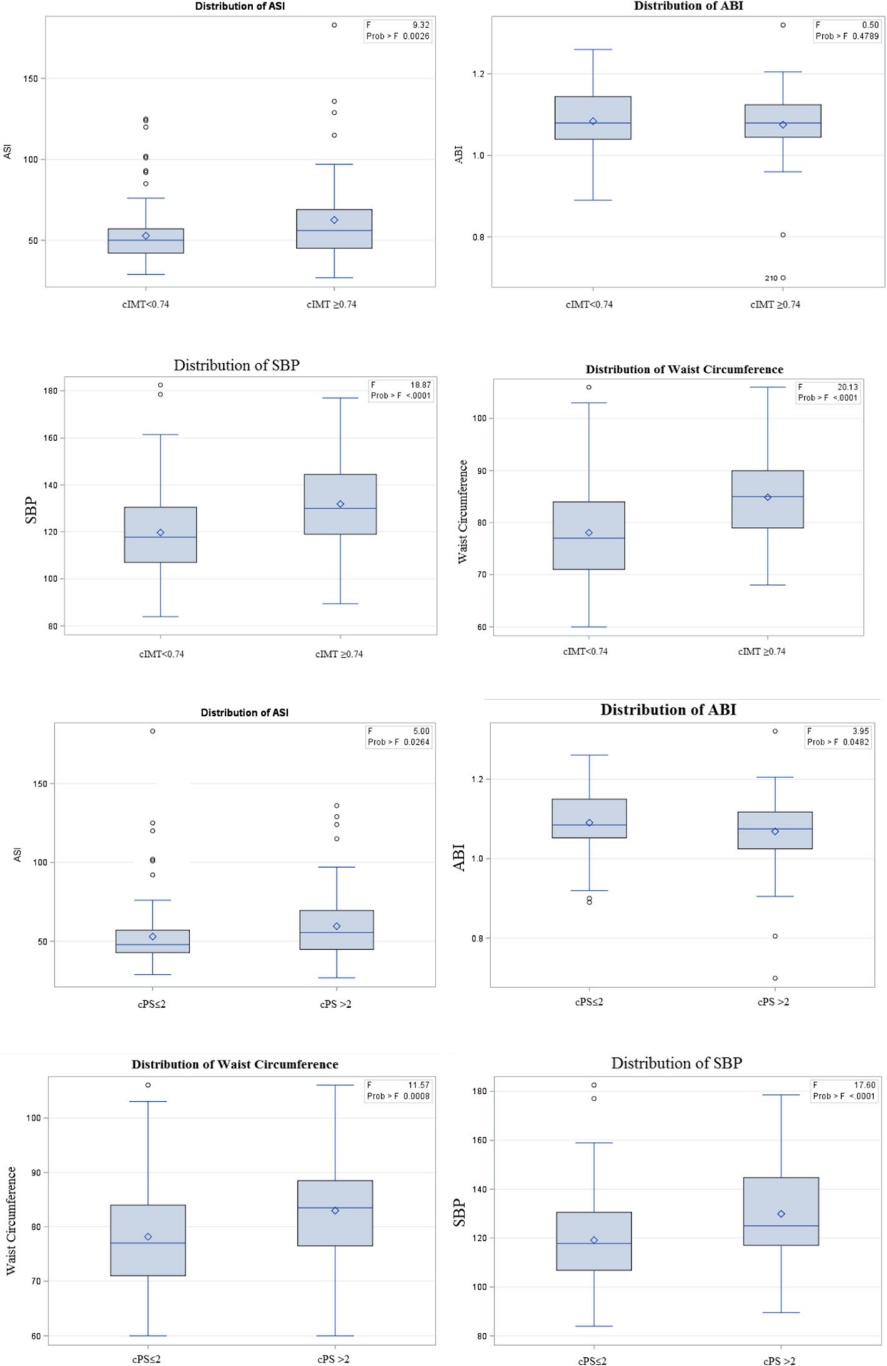
Distributions of different study variables between high and low cIMT and cPS. F statistics and *p* values of ANOVA test has been shown regarding each variable

Among 543 individuals, data of 212 individuals were entered in our study after examining the inclusion and exclusion criteria. They consisted of 98 men (46.2%) and 114 women (53.8%). Their mean age was 57.7 years, and the mean BMI was 23.81 kg/cm^2^. Nineteen (8.96%) had T2DM, and 50 individuals (23.58%) were stressed. Demographic characteristics of the study subjects are presented in Table 1 and Fig. 2, which also contains laboratory and physical exam results and a summary of duplex records and ASI and ABI measurements.

**Table 1:** Demographic characteristics, and laboratory, physical exam, and carotid ultrasound results of community-dwelling individuals, stratified by sex and age groups

Variable	*N*	Mean ± SD Number (%)	Age group (years)				Sex		
			< 45	45~60	> 60	*p*	Male	Female	*p*
Total	212	–	22 (10.4)	93 (43.9)	97 (45.7)	< 0.001	98 (46.2)	114 (53.8)	0.30
Age (years)	212	57.7 ± 10.4	37.9 ± 4.5	53.06 ± 3.83	66.73 ± 5.38	< 0.001	60.34 ± 11.14	55.51 ± 9.30	< 0.001
BMI (kg/cm^2^)	212	23.81 ± 3.17	24.17 ± 3.26	23.36 ± 3.51	24.16 ± 2.76	0.188	24.43 ± 2.99	23.28 ± 3.23	0.008
BMR	210	1230.45 ± 227.75	1292 ± 244.11	1188.94 ± 224.74	1256.49 ± 222.41	< 0.001	1422.88 ± 167.36	1062.07 ± 109.03	< 0.001
BC (cm)	211	94.54 ± 6.65	95.5 ± 8.46	93.68 ± 6.62	95.16 ± 6.17	0.240	96.04 ± 5.82	93.27 ± 7.05	0.002
WC (cm)	211	79.81 ± 9.97	79.22 ± 10.54	77.15 ± 9.85	82.53 ± 9.31	< 0.001	85.98 ± 8.54	74.57 ± 7.9	< 0.001
BF (%)	210	27.63 ± 6.89	29.23 ± 6.71	28.10 ± 7.04	26.84 ± 6.75	0.245	23.79 ± 5	30.99 ± 6.57	< 0.001
DM	212	19 (8.96)	1 (5.26)	5 (26.32)	13 (68.42)	0.112	13 (68.42)	6 (31.58)	0.053
HTN	212	56 (26.42)	1 (1.79)	17 (30.36)	38 (67.86)	< 0.001	32 (57.14)	24 (42.86)	0.062
SMK	212	46 (21.7)	3 (6.52)	16 (34.78)	27 (58.7)	0.127	44 (95.65)	2 (4.35)	< 0.001
Alc	211	25 (11.85)	4 (16)	10 (40)	11 (44)	0.675	21 (84)	4 (16)	< 0.001
Stressed	212	50 (23.58)	12 (24)	23 (46)	15 (30)	< 0.001	22 (44)	28 (56)	0.747
SBP (mmHg)	212	122.76 ± 18.36	111.45 ± 9.97	119.23 ± 18.08	128.71 ± 18.14	< 0.001	127.07 ± 16.03	119.05 ± 19.47	0.001
DBP (mmHg)	212	78.87 ± 10.38	74.61 ± 6.98	79.04 ± 11.44	79.68 ± 9.79	0.115	80.93 ± 9.97	77.1 ± 10.44	0.007
Pulse (beats/min)	212	69.86 ± 9.29	73.88 ± 6.87	71.24 ± 9.12	67.62 ± 9.43	0.002	69.71 ± 10.04	69.99 ± 8.63	0.826
FBS (mg/dl)	212	96.43 ± 21.98	91.09 ± 10.4	94.96 ± 18.15	99.05 ± 26.58	0.214	98.13 ± 26.95	94.97 ± 16.54	0.297
URCA (mg/dl)	212	5.62 ± 1.38	5.71 ± 1.44	5.62 ± 1.33	5.61 ± 1.41	0.95	5.75 ± 1.49	5.51 ± 1.26	0.2
BUN (mg/dl)	212	14.11 ± 4.36	14.63 ± 3.44	13.86 ± 4.43	14.23 ± 4.49	0.703	14.44 ± 4.26	13.82 ± 4.43	0.29
Cr (mg/dl)	212	0.96 ± 0.73	1.4 ± 2.15	0.89 ± 0.22	0.92 ± 0.26	0.01	1.02 ± 1.04	0.90 ± 0.26	0.238
AST (units/L)	212	23.69 ± 7.5	21.72 ± 4.65	24.3 ± 8.68	23.56 ± 6.73	0.343	23.46 ± 7.78	23.89 ± 7.28	0.681
ALT (units/L)	212	23.37 ± 14.34	21.36 ± 6.44	24.29 ± 16.76	22.95 ± 13.09	0.641	23.17 ± 11.39	23.55 ± 16.5	0.84
TGL (mg/dl)	212	118.79 ± 73.41	109.13 ± 63.99	113.48 ± 67.46	126.08 ± 80.53	0.403	126.14 ± 83.5	112.48 ± 63.17	0.177
Chol (mg/dl)	212	210.56 ± 35.84	200.95 ± 39.42	213 ± 35.91	210.41 ± 34.93	0.367	202.65 ± 31.99	217.36 ± 37.66	0.002
LDL (mg/dl)	212	136.30 ± 31.80	131.22 ± 34.67	136.48 ± 34.40	137.29 ± 28.62	0.721	134.71 ± 28.43	137.67 ± 34.5	0.5
HDL (mg/dl)	212	50.04 ± 14.42	49.31 ± 15.61	52.56 ± 15.15	47.79 ± 13.12	0.071	43.89 ± 13.01	55.33 ± 13.49	< 0.001
CRP (mg/L)	212	0.21 ± 0.75	0.1 ± 0.06	0.14 ± 0.19	0.3 ± 1.09	0.280	0.23 ± 1	0.19 ± 0.44	0.735
HBA1C (%)	212	5.80 ± 1.09	5.57 ± 0.45	5.67 ± 0.90	5.97 ± 1.31	0.096	5.87 ± 1.32	5.74 ± 0.83	0.376
Mean flow (ml)	212	224.11 ± 38.91	240.53 ± 43.22	228.64 ± 41.06	216.05 ± 34	0.008	227.69 ± 39.29	221.04 ± 38.48	0.215
Mean RI	203	0.65 ± 0.04	0.64 ± 0.03	0.64 ± 0.03	0.67 ± 0.04	< 0.001	0.67 ± 0.04	0.64 ± 0.04	< 0.001
Mean ECA DIA (cm)	212	0.36 ± 0.03	0.35 ± 0.03	0.35 ± 0.03	0.37 ± 0.03	< 0.001	0.37 ± 0.02	0.34 ± 0.03	< 0.001
Mean ICA DIA (cm)	212	0.43 ± 0.02	0.41 ± 0.02	0.42 ± 0.02	0.44 ± 0.02	< 0.001	0.43 ± 0.02	0.42 ± 0.02	< 0.001
Mean CCA DIA (cm)	212	0.57 ± 0.06	0.55 ± 0.05	0.55 ± 0.04	0.6 ± 0.06	< 0.001	0.60 ± 0.05	0.55 ± 0.05	< 0.001
Mean VA DIA (cm)	210	0.313 ± 0.02	0.3 ± 0.02	0.3 ± 0.02	0.31 ± 0.03	0.066	0.32 ± 0.02	0.3 ± 0.02	< 0.001
cIMT	208	0.68 ± 0.11	0.58 ± 0.06	0.63 ± 0.08	0.74 ± 0.10	< 0.001	0.71 ± 0.11	0.65 ± 0.09	< 0.001
cIMT > 0.74	208	54 (25.96)	0	9 (16.67)	45 (83.33)	< 0.001	36 (66.67)	18 (33.33)	< 0.001
cPS	212	2.19 ± 2.72	0.4 ± 1.05	1.3 ± 1.66	3.46 ± 3.19	< 0.001	2.77 ± 2.95	1.7 ± 2.4	0.003
Plaque presence	212	72 (33.96)	2 (2.78)	18 (25)	52 (72.22)	< 0.001	43 (59.72)	29 (40.28)	0.005
ASI	212	55.16 ± 20.51	47.9 ± 11.1	52.04 ± 17.07	59.79 ± 23.99	0.006	54.17 ± 15.66	56 ± 23.95	0.517
ABI	212	1.08 ± 0.07	1.07 ± 0.07	1.08 ± 0.06	1.08 ± 0.08	0.675	1.08 ± 0.07	1.07 ± 0.07	0.236
ABI < 0.95	212	9 (4.25)	1 (11.11)	3 (33.33)	5 (55.56)	0.795	3 (33.33)	6 (66.67)	0.509

*SD* standard deviation, *WC* waist circumference, *BC* bottom circumference, *BMI* body-mass index, *BMR* basal metabolic rate, *DM* diabetes mellitus, *HTN* hypertension, *SMK* smoking, *Alc* alcohol consumption, *BF* body fat, *Stressed* having high stress, *SBP* systolic blood pressure, *DBP* diastolic blood pressure, *FBS* fasting blood sugar, *URCA* uric acid level, *BUN* blood urea nitrogen, *Cr* creatinine, *AST* aspartate aminotransferase, *ALT* alanine aminotransferase, *TGL* triglycerides, *Chol* cholesterol, *LDL* low-density lipoprotein, *HDL* high-density lipoprotein, *CRP* C-reactive protein, *HBA1c* glycated hemoglobin, *cIMT* carotid intima media thickness, *cPS* carotid plaque score, *RI* resistance index, *ECA* external carotid artery, *ICA* internal carotid artery, *CCA* common carotid artery, *VA* vertebral artery, *DIA* diameter, *ASI* arterial stiffness index, *ABI* ankle brachial index, *p p* value

To determine the effects of different parameters, whether demographic or clinical, a Pearson correlation analysis was run on the study parameters. Only significant ones are shown in Table 2. Parameters not mentioned in Table 2 or indicated as being non-significant (ns) had no significant correlation with the cIMT, cPS, ASI and ABI.

**Table 2:** Statistically significant Pearson’s correlation coefficients (*p* value< 0.05) of carotid ultrasound parameters, the arterial stiffness index (ASI) and Ankle Brachial Index (ABI), with study parameters in community-dwelling individuals

Variable	cIMT		cPS		ASI		ABI	
	*r*	*p*	*r*	*p*	*r*	*p*	*r*	*p*
cIMT	1	–	0.53	< 0.001	0.20	0.002	ns	ns
cPS	0.53	< 0.001	1	–	0.16	0.014	−0.17	0.008
ASI	0.20	0.002	0.16	0.014	1	–	ns	ns
ABI	ns	ns	−0.17	0.008	ns	ns	1	–
Age	0.59	< 0.001	0.52	< 0.001	0.22	0.001	ns	ns
BC	0.18	0.007	ns	ns	ns	ns	ns	ns
BMI	0.22	0.001	ns	ns	ns	ns	ns	ns
BMR	0.19	0.005	ns	ns	ns	ns	ns	ns
WC	0.35	< 0.001	0.20	0.002	ns	ns	ns	ns
BF	ns	ns	ns	ns	ns	ns	−0.13	0.049
Stressed	0.19	0.005	ns	ns	ns	ns	ns	ns
SBP	0.37	< 0.001	0.31	< 0.001	0.48	< 0.001	ns	ns
DBP	0.18	0.008	ns	ns	0.30	< 0.001	0.14	0.038
Pulse	−0.22	0.001	−0.14	0.035	− 0.20	0.002	− 0.14	0.040
URCA	ns	ns	ns	ns	ns	ns	ns	ns
BUN	ns	ns	ns	ns	ns	ns	ns	ns
CRP	0.23	< 0.001	ns	ns	ns	ns	ns	ns
Mean flow	−0.23	< 0.001	−0.22	0.001	ns	ns	ns	ns
Mean RI	0.26	< 0.001	0.28	< 0.001	0.25	< 00.1	−0.14	0.034
ECA DIA	0.16	0.017	0.13	0.047	0.13	0.043	ns	ns
ICA DIA	0.39	< 0.001	0.14	0.040	ns	ns	ns	ns
CCA DIA	0.4	< 0.001	0.32	< 0.001	0.21	0.001	ns	ns
VA DIA	ns	ns	ns	ns	ns	ns	0.14	0.039

*cIMT* carotid intima media thickness, *cPS* carotid plaque score, *ABI* ankle brachial index, *RI* resistance index, *ECA* external carotid artery, *ICA* internal carotid artery, *CCA* common carotid artery; VA vertebral artery, *DIA* diameter, *WC* waist circumference, *BC* bottom circumference, *BMI* body-mass index, *BMR* basal metabolic rate, *BF* body fat, *Stressed* having high stress, *SBP* systolic blood pressure, *DBP* diastolic blood pressure, *FBS* fasting blood sugar, *URCA* uric acid level, *BUN* blood urea nitrogen, *CRP* C-reactive protein, *ns* not significant, *p* p value, *r* Pearson correlation coefficient

To determine the effects of the variables with significant correlations with the cIMT and cPS, a multivariate analysis was done by running a linear regression model for the cIMT and cPS. As the cIMT was not normally distributed (Shapiro-Wilk wildstatistics = 0.96, *p* < 0.001) a decimal logarithm (log 10 basis) was taken of cIMT (Shapiro-Wilk wild statistics = 0.98, *p* = 0.104). Results are given in Table 3.

**Table 3:** Linear regression models to predict the carotid intima media thickness (cIMT) and carotid plaque score (cPS) and logistic regression models to predict thickened carotid intima media and a high cPS

Variable	Log cIMT		cPS		cIMT ≥0.74		cPS > 2	
	B	*p*	B	*p*	OR	95% CI	OR	95% CI
Age	**0.006**	**< 0.001**	**0.104**	**< 0.001**	**1.13**	**1.08~1.19**	**1.12**	**1.07~1.16**
Gender (male)	−0.005	0.823	0.412	0.283	–	–	–	–
WC	**0.002**	**0.015**	−0.012	0.549	**1.04**	**1.00~1.09**	–	–
DM	0.032	0.312	0.897	0.136	–	–	–	–
HTN	−0.0050	0.807	**1.12**	**0.008**	–	–	**2.57**	**1.26~5.24**
SMK	0.007	0.750	–	–	–	–	–	–
Stressed	−0.016	0.412	–	–	–	–	–	–
SBP	**0.001**	**0.005**	0.014	0.146	1.02	0.99~1.04	**–**	**–**
Pulse	−0.001	0.118	−0.013	0.435	–	–	–	–
CRP	**0.034**	**0.001**	–	–	1.39	0.75~2.58	–	–

*WC* waist circumference, *DM* diabetes mellitus, *HTN* hypertension, *SMK* smoking, *Stressed* having high stress, *SBP* systolic blood pressure, *DBP* diastolic blood pressure, *CRP* C-reactive protein, − means not included in the model, *B* Beta coefficient, *p* p value, *OR* Odds Ratio. Statistically significant Beta coefficients (*p* < 0.05) and Odds Ratios are in bold

The ASI was correlated with cIMT with *r* = 0.2; so after adjusting for age, this correlation disappeared (B = 0.0006, *p* = 0.164, *R*^2^ = 0.37). Regarding the relationship of ASI and cPS, Pearson’s correlation was 0.16. Again after adjusting for age, the relationship disappeared (B = 0.006, *p* = 0.390). So relationships of ASI with the cIMT and cPS, had a confounding effect with age. The ABI was correlated with cPS at *r* = − 0.17, and this negative correlation stayed robust after adjusting for age, sex, and HTN (B = − 4.10, *p* = 0.044).

To calculate the ORs of determinants of cIMT and cPS, a logistic regression analysis was run and the 95% CI was calculated for determining cIMT ≥0.74 and cPS > 2 (third quartile) as can be seen in Table 3. The odds of having a thick cIMT increased by 13% with every year increase in age and by 4% with every centimeter increase in the WC. The odds of having a high cPS was 2.57 times greater in people with HTN, and it increased by 12% with each year increase in age.

Power calculations for Pearson’s correlations with alpha error of 0.05 for cIMT and ASI (*r* = 0.2) was 90.2%. Power for cPS and ASI (*r* = 0.16) was 75.6% and power for cPS and ABI (*r* = − 0.17) was calculated 80%.

## Discussion

Carotid duplex parameters, certain laboratory tests, lifestyle parameters, and two arterial stiffness indexes were examined in 212 individuals from the general population. Only age, WC, SBP, and CRP had significant independent determinant effects on the cIMT, while only age and a history of HTN affected the cPS. The ASI could not determine the cIMT, although it was correlated with it. The ABI was independently associated with the cPS.

Among the proposed techniques of detecting subclinical atherosclerosis, we evaluated the cIMT and ASI, as well as the biomarkers LDL, HDL, Chol, CRP, TGL, HBA1c, and creatinine. We used cIMT as a method of measuring subclinical atherosclerosis, to test the association of atherosclerosis with ASI. The ASI was correlated with both cIMT and cPS; however it was not independent of age. Kao et al. [2] concluded from their study that the ASI can be predictive of atherosclerosis as it was correlated with the cIMT in healthy individuals, independent of other factors. Our results contradicted their results, as the correlation between the ASI and cIMT was not robust. Our study had a larger sample size, and results of their studies were more likely to be biased.

Brasileiro et al. [16] in a study of 118 diabetic patients found a Pearson’s correlation of − 0.23 between the ABI and cIMT. Although our results did not show a correlation between the ABI and cIMT, we found a stable correlation between the ABI and cPS, another marker of atherosclerosis.

Hernandez et al. [17] found no association between the ABI and perceived stress levels. Their data are in accordance with our results. Kim et al. [18] found no correlation between perceived stress and the cIMT. And even though we found a relationship between them, after adjusting for other variables, it disappeared. So our results confirm those of Kim et al. Chronic stress might have an effect on the cIMT, as Roepke et al. [19] suggested. But we only measured a very simple self-perceived stress level based on one question, and so it cannot be a valid tool for acute or chronic stress.

The WC can reflect subclinical atherosclerosis and can also predict atherosclerosis progression [20]. Shen et al. [21] in a study of 2365 women concluded that WC can be an independent risk factor of the cIMT. We also found that WC was the only independent determinant of cIMT besides age.

Liu et al. [22] found that cPS was not correlated with age and DBP, but it was correlated with SBP. We found that males tended to have a higher cPS. But other published results are in accordance with our study. Balashenko et al. [23] in a study of 155 individuals found that the cPS was higher in people with HTN. We also found HTN to be an independent determinant of a high cPS. Some studies showed that subjects with HTN had a higher cIMT. [24, 25] We also found that the SBP was an independently associated with the cIMT. Elevated blood pressure can cause endothelial damage and atherosclerosis by increasing shear stress, enhancing the vascular tone, and activating the sympathetic nervous system and renin-angiotensin aldosterone system [26].

T2DM was shown to be correlated with different stages of subclinical atherosclerosis and the ABI [24]. Although our results showed this correlation, we did not find that T2DM was an independent risk factor for subclinical atherosclerosis or the ABI, although the limited number of T2DM subjects in our study makes any kind of speculation very difficult.

Eltoft et al. [26] reported that the CRP level can be a determinant of atherosclerosis and not plaque formation. Our results are in line with that study, as we found that CRP was an independent factor influencing the cIMT. Although Khera et al. [27] found that an association of CRP and subclinical atherosclerosis was dependent on other risk factors, they concluded that CRP is a poor predictor of atherosclerosis.

Although some studies found that URCA was an independent risk factor for subclinical atherosclerosis [28], other studies are in opposition to these results [29]. Our study results found that URCA was not correlated with cIMT or cPS.

## Limitations

This study had certain limitations. Limited sample size was considered as an important one. Statistical power for determination of Pearson’s correlation between cPS and ASI had not reached 80% and could be considered in further studies with more sample size. Besides, we only included healthy community dwelling individuals which makes predictions regarding atherosclerosis in certain groups of patients like those with stable angina or other cardiovascular diseases difficult. Studies with more sample size and different atherosclerotic detection methods such as Pulse Wave Velocity (PWV) on various CVD patients are recommended. We should also mention that baseline characteristics of individuals could account for the observed results in our study. Although we controlled for baseline characteristics in our multivariate analysis, matched case control studies could be considered in the future studies.

## Conclusions

The WC, age, CRP level, and SBP were independently associated with cIMT, and an HTN history and age could be associated with the cPS. Only age and WC and only age and HTN history could be respectively associated with a high cIMT and cPS. Public health policies such as weight reduction strategies to help individuals reduce their waist circumference and treating hypertension as early as possible, seems to be essential in order to control subclinical atherosclerosis.

The use of the ASI cannot replace carotid ultrasound in detecting subclinical atherosclerosis because it is not independently associated with high cIMT and cPS while ABI can be used in detection of high cPS in healthy community-dwelling individuals. Models consist of age, body compositions like waist circumference and hypertension history can be used in further assessment of atherosclerosis.

## Abbreviations

ANOVA: Analysis of variance
BMI: Body Mass Index
BUN: Blood Urea Nitrogen
CCA: Common Carotid Artery
CHOL: Cholesterol
cIMT: Carotid intima media thickness
cPS: Carotid Plaque Score
CV: Cardiovascular
CVD: Cardio Vascular Disease
DBP: Diastolic Blood Pressure
ECA: External Carotid Artery
FBS: Fasting blood sugar
HBA1c: glycated hemoglobin
HDL: High-density lipoprotein
HTN: Hypertension
ICA: Internal Carotid Artery
LDL: Low-density lipoprotein
Log: Logarithm with base 10
RI: Resistance Index
SBP: Systolic Blood Pressure
SD: Standard Deviation
T2DM: Type 2 Diabetes Mellitus
URCA: Uric Acid Level
VA: Vertebral Artery
WC: waist circumference
WC: waist circumference
